# Large-scale preparation of nanoporous TiO_2_ film on titanium substrate with improved photoelectrochemical performance

**DOI:** 10.1186/1556-276X-9-190

**Published:** 2014-04-24

**Authors:** Beihui Tan, Yue Zhang, Mingce Long

**Affiliations:** 1 School of Environmental Science and Engineering, Shanghai Jiao Tong University, Dongchuan Road 800, Shanghai 200240, China

**Keywords:** Nanoporous TiO_2_ film, Titanium substrate, Photocurrent, Photoelectrocatalysis

## Abstract

Fabrication of three-dimensional TiO_2_ films on Ti substrates is one important strategy to obtain efficient electrodes for energy conversion and environmental applications. In this work, we found that hierarchical porous TiO_2_ film can be prepared by treating H_2_O_2_ pre-oxidized Ti substrate in TiCl_3_ solution followed by calcinations. The formation process is a combination of the corrosion of Ti substrate and the oxidation hydrolysis of TiCl_3_. According to the characterizations by scanning electron microscopy (SEM), X-ray diffraction (XRD), and diffuse reflectance spectroscopy (DRS), the anatase phase TiO_2_ films show porous morphology with the smallest diameter of 20 nm and possess enhanced optical absorption properties. Using the porous film as a working electrode, we found that it displays efficient activity for photoelectrocatalytic decolorization of rhodamine B (RhB) and photocurrent generation, with a photocurrent density as high as 1.2 mA/cm^2^. It represents a potential method to fabricate large-area nanoporous TiO_2_ film on Ti substrate due to the scalability of such chemical oxidation process.

## Background

In recent years, TiO_2_ has been widely studied and applied in diverse fields, such as photocatalysis, dye-sensitized solar cell, self-cleaning surface, sensor, and biomedicine [1-6]. It is well known that TiO_2_ nanoparticles have the potential to remove recalcitrant organic pollutants in wastewater. However, it is prerequisite to produce immobilized TiO_2_ photocatalysts with highly efficient activity by scale-up methods. Recently, considerable efforts have been taken to use metallic titanium as the precursor to develop three-dimensional TiO_2_ films with controllable ordered morphologies, such as nanotubes [7], nanorods [8], nanowires [9], and nanopores [10]. The *in situ*-generated TiO_2_ films over titanium substrates possess such advantages as stable with low carbon residual, excellent mechanical strength, and well electron conductivity, which make them suitable to be used as electrodes for photoelectrochemical-related applications [6,11]. Although a well-defined structural nanotube or nanoporous TiO_2_ film on metallic Ti can be synthesized by an anodic method [6,7,10-13], it is still a big challenge to scale up the production of such TiO_2_ film due to the limitation of electrochemical reactor and the high energy consumption. Chemical oxidation methods by treating titanium substrates in oxidation solutions are more scalable for various applications. By soaking titanium substrates in H_2_O_2_ solution followed with calcinations, titania nanorod or nanoflower films can be obtained [8,14]. However, the film always displays discontinuous structure with many cracks, and its thickness is less than 1 μm [8,15]. Both of these would result in a low photoelectrochemical performance. With the addition of concentrated NaOH in the H_2_O_2_ solution, a porous nanowire TiO_2_ film can be achieved after an ionic exchange with protons and subsequent calcinations [9]. Employing NaOH and organic solvent as the oxidation solution and elevating the treating temperature, Ti substrate would completely transform into free-standing TiO_2_ nanowire membranes [16]. However, the disappearance of Ti substrate makes this membrane impossible to serve as an electrode.

Compared to titanium alkoxides or TiCl_4_, there are much fewer reports on the synthesis of TiO_2_ nanostructure with the precursor of TiCl_3_. Normally, anatase TiO_2_ film can be fabricated via the anodic oxidation hydrolysis of TiCl_3_ solution [17,18]. Recently, Hosono et al. synthesized rectangular parallelepiped rutile TiO_2_ films by hydrothermally treating TiCl_3_ solution with the addition of a high concentration of NaCl [19], and Feng et al. developed TiO_2_ nanorod films with switchable superhydrophobicity/superhydrophilicity transition properties via a similar method [20]. Moreover, a hierarchically branched TiO_2_ nanorod film with efficient photon-to-current conversion efficiency can be achieved by treating the nanorod TiO_2_ film in TiCl_3_ solution [21]. However, all of these nanostructural TiO_2_ films from TiCl_3_ solution were grown over glass or alumina substrates. Fabricating nanostructral TiO_2_ films over metallic Ti substrates is a promising way to providing high-performance photoresponsible electrodes for photoelectrochemical applications. The obstacle for starting from Ti substrates and TiCl_3_ solution must be the corrosion of metallic Ti at high temperatures in the HCl solution, which is one of the components in TiCl_3_ solution. However, the corrosion could also be controlled and utilized for the formation of porous structures. According to reports, the general method to prepare nanoporous TiO_2_ film on Ti substrate is through anodic oxidation and post-sonication [10,12]. In this contribution, we proposed a facile way to fabricate nanoporous TiO_2_ films by post-treating the H_2_O_2_-oxidized TiO_2_ film in a TiCl_3_ solution. The as-prepared nanoporous TiO_2_ film display homogeneous porous structure with enhanced optical adsorption property and photoelectrocatalytic performance, which indicates that the film is promising in the applications of water purification and photoelectrochemical devices.

## Methods

Cleansed Ti plates (99.5% in purity, Baoji Ronghao Ti Co. Ltd., Shanxi, China) with sizes of 1.5 × 1.5 cm^2^ were pickled in a 5 wt% oxalic acid solution at 100°C for 2 h, followed by rinsing with deionized water and drying in an air stream. The nanoporous TiO_2_ film was prepared by a two-step oxidation procedure. Briefly, the pretreated Ti plate was firstly soaked in a 15 mL 20 wt% H_2_O_2_ solution in a tightly closed bottle, which was maintained at 80°C for 12 h. The treated Ti plate was rinsed gently with deionized water and dried. Then, it was immersed in a 10 mL TiCl_3_ solution (0.15 wt%) at 80°C for 2 h. Finally, the film was cleaned, dried, and calcined at 450°C for 2 h. The obtained nanoporous TiO_2_ film was designed as NP-TiO_2_. Two control samples were synthesized, including the one designed as TiO_2_-1, which was obtained by directly calcining the cleansed Ti plate, and the other named as TiO_2_-2, which was prepared by one-step treatment of the Ti plate in a TiCl_3_ solution.

The surface morphology of TiO_2_ films was observed using a field emission scanning electron microscope (SEM; Zeiss Ultra 55, Oberkochen, Germany). The crystal phases were analyzed using a powder X-ray diffractometer (XRD; D8 Advance, Bruker, Ettlingen, Germany) with Cu Kα radiation, operated at 40 kV and 36 mA (*λ* = 0.154056 nm). UV-vis diffuse reflectance spectra (DRS) were recorded on a Lambda 950 UV/Vis spectrophotometer (PerkinElmer Instrument Co. Ltd., Waltham, MA, USA) and converted from reflection to absorption by the Kubelka-Munk method.

Photoelectrochemical test systems were composed of a CHI 600D electrochemistry potentiostat, a 500-W xenon lamp, and a homemade three-electrode cell using as-prepared TiO_2_ films, platinum wire, and a Ag/AgCl as the working electrode, counter electrode, and reference electrode, respectively. A 0.5 M Na_2_SO_4_ solution purged with nitrogen was used as electrolyte for all of the measurements.

The photocatalytic or photoelectrocatalytic degradation of rhodamine B (RhB) over the NP-TiO_2_ film was carried out in a quartz glass cuvette containing 20 mL of RhB solution (C_28_H_31_ClN_2_O_3_, initial concentration 5 mg/L). The pH of the solution was buffered to 7.0 by 0.1 M phosphate. The solution was stirred continuously by a magnetic stirrer. Photoelectrocatalytic reaction was performed in a three-electrode system with a 0.5-V anodic bias. The exposed area of the electrodes under illumination was 1.5 cm^2^. Concentration of RhB was measured by spectrometer at the wavelength of 554 nm.

## Results and discussion

**Figure 1:**
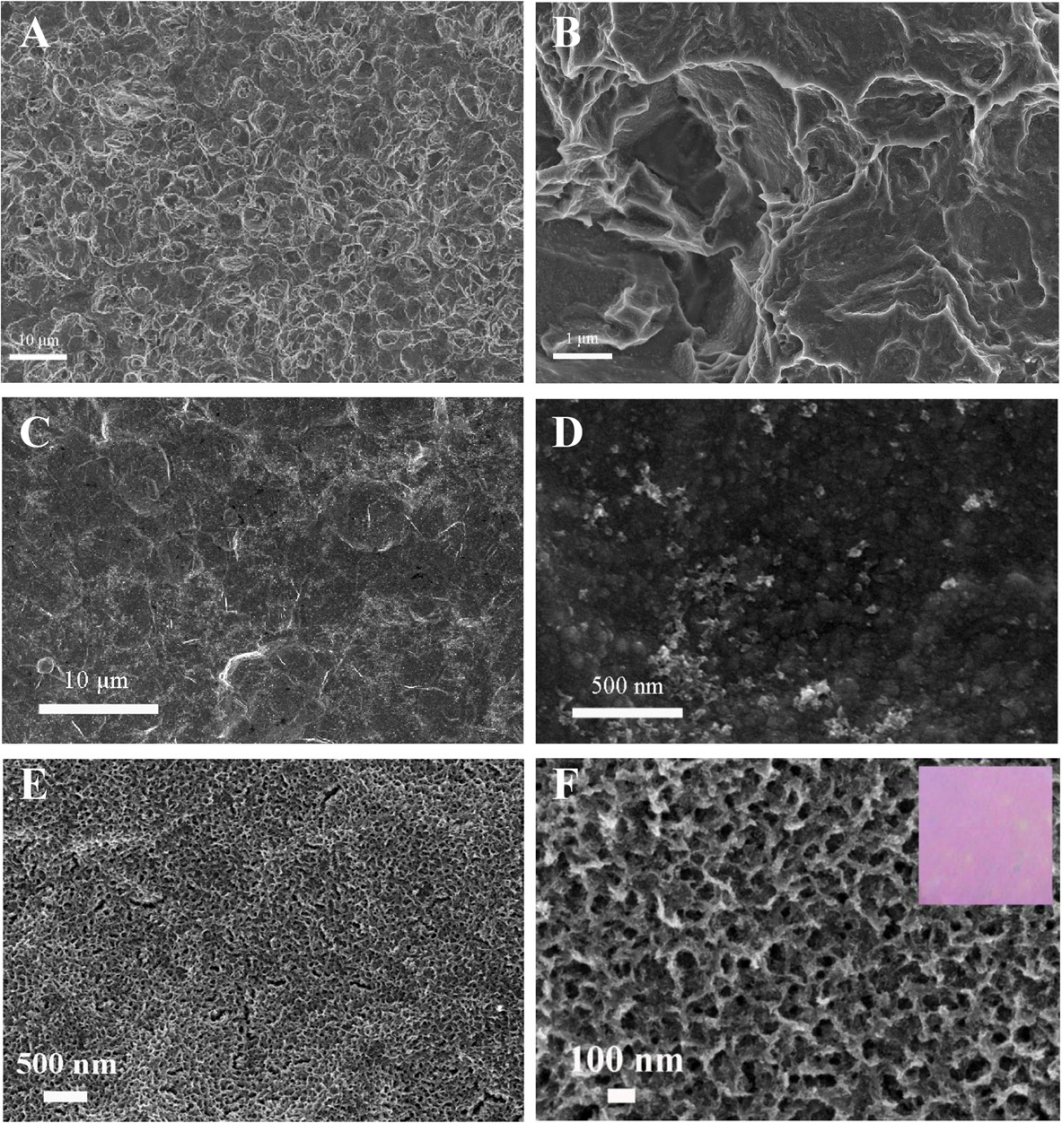
**FE-SEM images of TiO**_**2 **_**films over Ti plates. (A, B)** TiO_2_-1, **(C, D)** TiO_2_-2, and **(E, F)** NP-TiO_2_ (the inset in **(F)** shows the digital picture of the NP-TiO_2_ film).

Figure 1 shows the surface morphologies of films obtained by different procedures. The control sample TiO_2_-1 is obtained by the calcination of the pickled Ti plate at 450°C for 2 h. The typical coarse surface formed from the corrosion of Ti plate in oxalic solution can be observed (Figure 1A,B). By oxidation at a high temperature, the surface layer of titanium plate transformed into TiO_2_. However, the surface morphology shows negligible change. The film of TiO_2_-2, which is synthesized by directly treating the cleansed and pickled Ti plate in TiCl_3_ solution, displays smoother surface with no observable nanostructure (Figure 1C,D). Moreover, there are discernible TiO_2_ particles dispersing over the surface. It suggests that in the TiCl_3_ solution the surface morphology of Ti plate has been modified after dissolution, precipitation and deposition processes. By treating the H_2_O_2_ pre-oxidized Ti plate in TiCl_3_, the film displays a large-scale irregular porous structure, as shown in Figure 1E,F. Moreover, the appearance of NP-TiO_2_ film is red color (as inset in Figure 1F), which is different from the normal appearance of most anodic TiO_2_ nanorod or nanotube films [22]. The pores are in the sizes of 50 to 100 nm on the surface and about 20 nm inside; the walls of the pores are in the sizes of 10 nm and show continuous connections. Such hierarchical porous structure contributes to a higher surface area of the TiO_2_ film. Normally, titanium suffers from corrosion in the hot HCl solution, and the corrosion rate depends on the temperature and the concentration of acid. Without pre-oxidation, the surface layer of Ti plate is exposed to be etched and dissolved in the reaction solution at a medium temperature. Simultaneously, the TiOH^2+^ and Ti(IV) polymer generated by the hydrolysis of TiCl_3_ would precipitate and deposit over the surface (Equations 1 and 2) so as to retard the corrosion of Ti plate and avoid the completed dissolution of Ti plate [17,19]. For the NP-TiO_2_ film, after the first step of oxidation in H_2_O_2_ solution, peroxo complexes coordinated to Ti(IV) have already formed, which cover most parts of the surface and be ready for further growth by the interaction with the oxidation hydrolytic products of TiCl_3_. However, it is also possible that HCl solution enters the interstitial of the TiO_2_ nanorod film and induces etching of the substrate Ti. At the experimental temperature, the dissolution of Ti is slow. With the reorganization of Ti(IV) polymer precursor, a porous structure forms over the Ti plate, as shown in Figure 1F.

(1) $${\mathrm{Ti}}^{3+}+H_{2}O\Leftrightarrow {\mathrm{TiOH}}^{2+}+H^{+}$$

(2) $$\begin{array}{l} {\mathrm{TiOH}}^{2+}+O_{2}\to \mathrm{Ti}\left(\mathrm{IV}\right)\;\mathrm{oxo}\;\mathrm{species}\\ \;+{O_{2}}^{-}\to {\mathrm{TiO}}_{2} \end{array}$$

**Figure 2:**
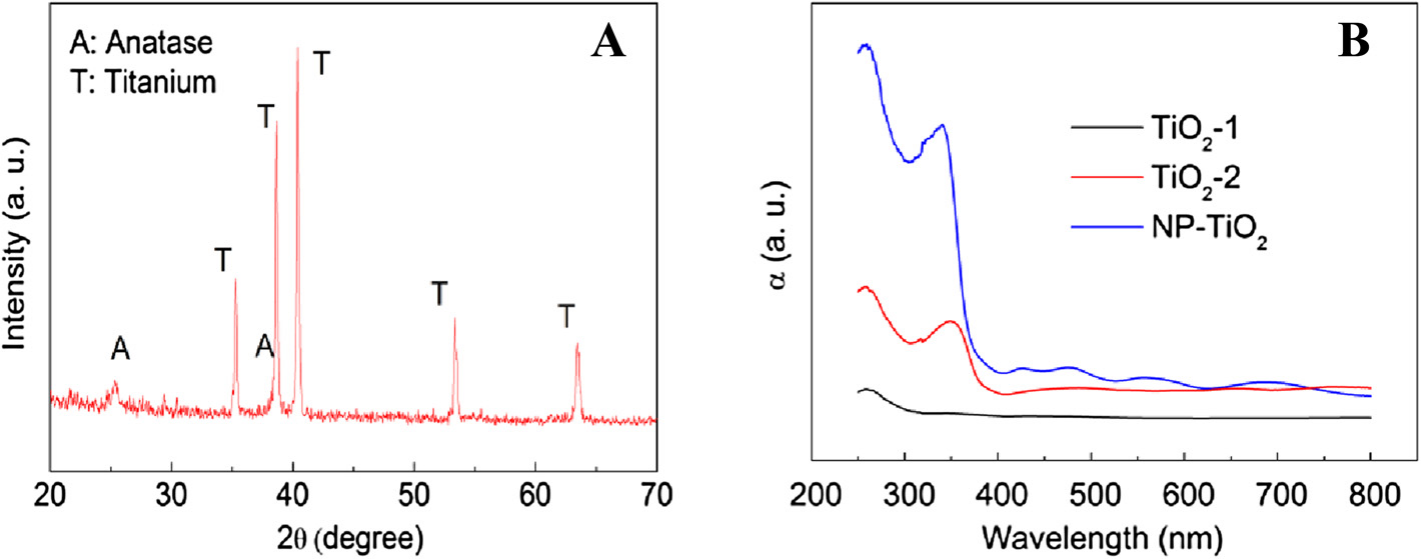
**XRD pattern of NP-TiO**_**2 **_**(A) and the DRS spectra of various films (B).**

Figure 2A is the XRD pattern of NP-TiO_2_ film. The strong diffraction peaks at about 35.2°, 38.7°, 40.4°, 53.3°, and 63.5° can be assigned to the metallic Ti (JCPDS 44-1294). At the same time, the peak at 25.3° corresponds to the (101) plane of anatase phase TiO_2_ (JCPDS 83-2243). Diffraction peaks of rutile or brookite cannot be found, indicating that the titania film is composed of exclusively anatase. DRS spectra were measured to analyze the optical absorption properties of the films, as shown in Figure 2B. There is almost no optical adsorption for the TiO_2_-1 film, indicating that only a very thin layer of metallic Ti transforms into TiO_2_ after the calcination of Ti plate, and this contributes a poor photoresponse performance. TiO_2_-2 film displays a typical semiconductor optical absorption with the adsorption edge at about 380 nm, corresponding to the band gap of anatase TiO_2_. However, the absorption is relatively low, indicating that only few of TiO_2_ nanoparticles deposit over the surface of TiO_2_-2 film. The strong optical absorption appearing below 400 nm for NP-TiO_2_ film suggests a full growth of TiO_2_ layer over the Ti plate. Moreover, several adsorption bands centered at about 480, 560, and 690 nm can be observed in the spectrum of NP-TiO_2_ film. They possibly originated from the periodic irregular nanoporous structure. Such nanoporous structure is favorable to increase the photoresponsible performance, because the incident light that entered the porous structure would extend the interaction of light with TiO_2_ and result in an enhanced absorption performance, which can be observed in other nanotube or photonic crystal structural TiO_2_ films [22,23].

**Figure 3:**
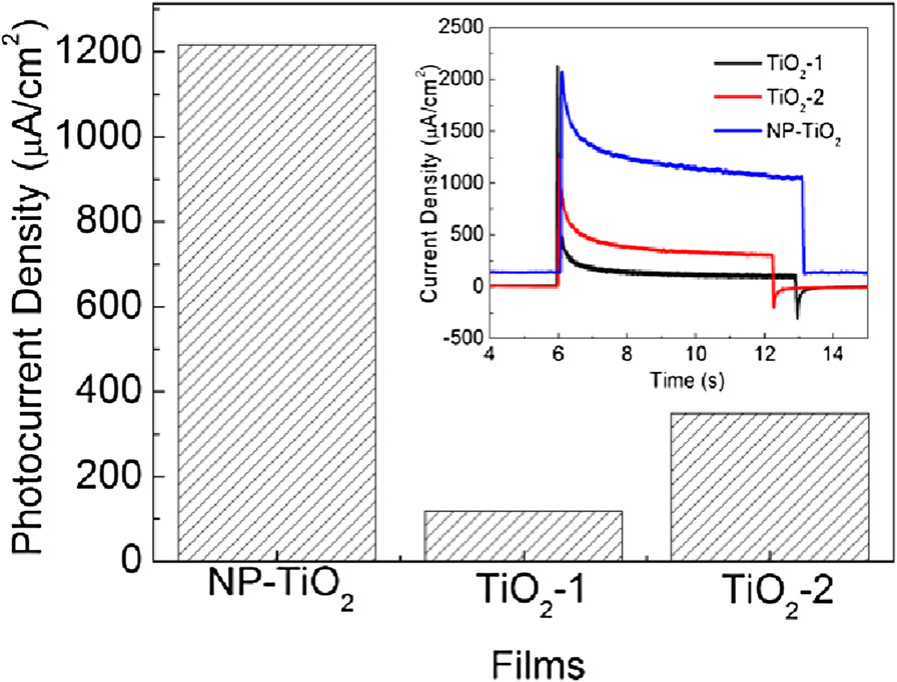
**A comparison of photocurrent density of various films.** The inset shows a comparison of the current transients (applied potential: 0.2 V vs. Ag/AgCl).

Using TiO_2_ films as the working electrodes in a three-electrode system, photocurrents under irradiation with full spectrum of light source were measured and compared, as shown in Figure 3. From the current transients (inset in Figure 3), all films show anodic photocurrents upon illumination, corresponding to the n-type photoresponse of TiO_2_. For TiO_2_-1 film, the initial anodic photocurrent spike is very strong and subsequently decays quickly. Simultaneously, a cathodic overshoot appears immediately when the light is switched off. The anodic current spike and cathodic overshoot are occasionally observed in many cases, and which is generally regarded as the indication of the surface recombination of photogenerated charges [24-26]. A decay of anodic current immediately after the initial rise of the signal when the light is switched on is attributed to photogenerated electron transfer to the holes trapped at the surface states or the intermediates which originated from the reaction of holes at the semiconductor surface. With the accumulation of the intermediates, the electrons are trapped by the surface states, resulting in an anodic current spike. Owing to the same reason, the intermediates or trapped holes would induce a cathodic overshoot when switching off the light. The obvious strong spike for the illuminated TiO_2_-1 film suggests the slow consumption of holes and the corresponding oxidation process, which is related to the activity of the surface TiO_2_ layer. The poor crystallinity, large TiO_2_ particles, and the small amount of TiO_2_ in the directly oxidized film would result in the poor photoelectrochemical performance. However, the transient of NP-TiO_2_ film is different, displaying much smaller anodic current spike and more stable photocurrent. The photocurrent density is calculated as the difference of the current density upon illumination at the center time and in the dark, which is shown as a graph in Figure 3. NP-TiO_2_ film possesses the highest photocurrent density, which is about 1.2 mA/cm^2^, significantly higher than the corresponding TiO_2_-1 and TiO_2_-2 films. The efficient photoelectrochemical performance can be attributed to the porous structure of NP-TiO_2_ film, in which the interaction time between TiO_2_ and light would be increased due to the trapped photons inside the pores, corresponding to its enhanced optical absorption.

**Figure 4:**
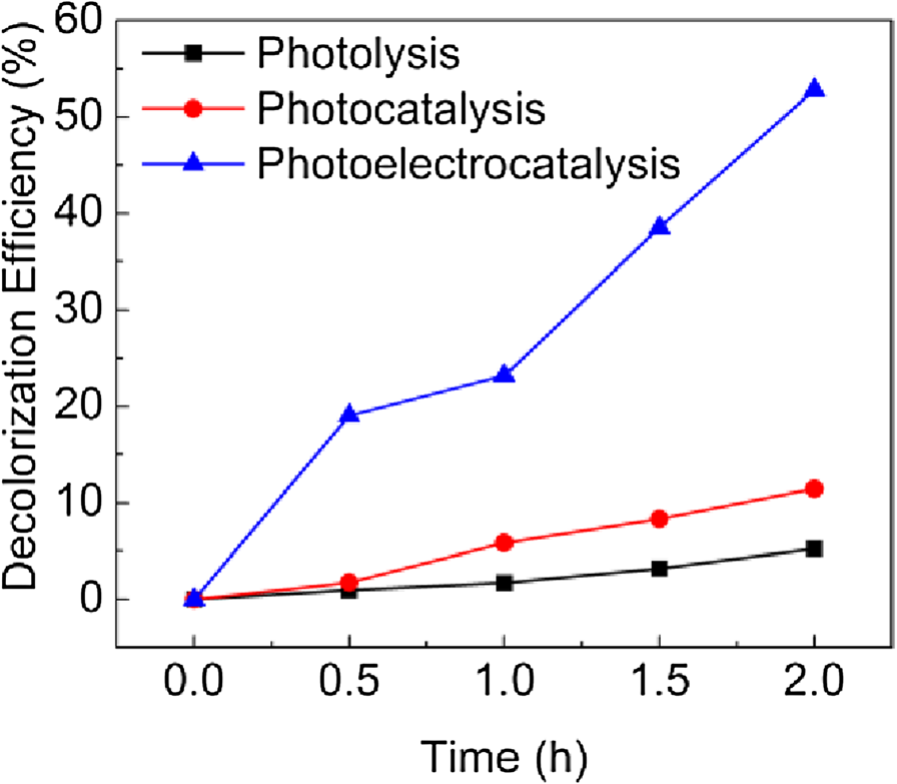
RhB decolorization as a function of time under various conditions.

The performance of the NP-TiO_2_ film was further tested by photoelectrocatalytic degradation of RhB solutions. The decolorization of RhB by photolysis is low, only 5.2% reduction observed after 2 h of irradiation (Figure 4). Without an applied bias, by illuminating the solution with the NP-TiO_2_ film, the decolorization efficiency only improved to about 11%. This low photocatalytic efficiency of the film could be attributed to the too small active area of the film and the phosphate in the buffered solution, which is regarded as the scavenger of radicals [27]. However, with a bias of 0.5 V vs. Ag/AgCl, the decolorization of RhB has been significantly improved, about 52.8% decolorization of RhB solution after 2 h of irradiation. Photoelectrocatalysis is a combination of photocatalysis and electrooxidation using the semiconductor films. By this method, an anodic bias on NP-TiO_2_ film is used to drive photogenerated electrons and holes moving toward different direction, so as to suppress the recombination and promote the organic degradation [11,28]. Moreover, besides the improved optical absorption, the porous structure also contributes to a short diffusion path for RhB molecules to the active surface area. Therefore the NP-TiO_2_ film displays efficient photoelectrocatalytic activity for organic degradation. It can be expected that the chemical oxidation method for NP-TiO_2_ films is scalable for practical applications. With a larger active area, the NP-TiO_2_ film is potential to be used as an efficient electrode for energy conversion and organic pollutant removal.

## Conclusions

A nanoporous TiO_2_ film on Ti substrate was synthesized by treating the initially H_2_O_2_-oxidized Ti plate in hot TiCl_3_ solution and followed by calcinations. The pre-oxidation in H_2_O_2_ solution is necessary to form such porous structure, indicating that the formation process is a combination of the corrosion of Ti substrate and the oxidation hydrolysis of TiCl_3_. The film possesses exclusively anatase phase and hierarchical porous morphology, with the diameter of the inside pores as small as 20 nm. The porous TiO_2_ film displays enhanced optical absorption, photocurrent generation, and efficient photoelectrocatalytic activity for RhB decolorization. The generated photocurrent density can reach as high as 1.2 mA/cm^2^. The chemical oxidation method for the nanoporous TiO_2_ film is possible to be scaled up and developed into a strategy to provide efficient TiO_2_ electrodes for diverse applications.

## Competing interests

The authors declare that they have no competing interests.

## Authors' contributions

ML designed the experiments. BT and YZ carried out all of the experiments. BT and ML wrote the paper. All authors read and approved the final manuscript.

## References

[B1] Fujishima A Zhang X Tryk DA TiO_2_ photocatalysis and related surface phenomena Surf Sci Rep 2008 9 515 582 10.1016/j.surfrep.2008.10.001

[B2] Tran PD Wong LH Barber J Loo JSC Recent advances in hybrid photocatalysts for solar fuel production Energ Environ Sci 2012 9 5902 10.1039/c2ee02849b

[B3] Kubacka A Fernandez-Garcia M Colon G Advanced nanoarchitectures for solar photocatalytic applications Chem Rev 2012 9 1555 1614 10.1021/cr100454n 22107071

[B4] Long MC Wu D Cai WM Photoinduced hydrophilic effect and its application on self-cleaning technology Recent Pat Eng 2010 9 189 199 10.2174/187221210794578619

[B5] Cheyne R Smith T Trembleau L McLaughlin A Synthesis and characterisation of biologically compatible TiO_2_ nanoparticles Nanoscale Res Lett 2011 9 1 6 10.1186/1556-276X-6-423 PMC3211840 21711954

[B6] Zheng Q Zhou BX Bai J Li LH Jin ZJ Zhang JL Li JH Liu YB Cai WM Zhu XY Self-organized TiO_2_ nanotube array sensor for the determination of chemical oxygen demand Adv Mater 2008 9 1044 1049 10.1002/adma.200701619

[B7] Macak JM Tsuchiya H Taveira L Aldabergerova S Schmuki P Smooth anodic TiO_2_ nanotubes Angew Chem Int Ed 2005 9 7463 7465 10.1002/anie.200502781 16247823

[B8] Wu JM Zhang TW Zeng YW Hayakawa S Tsuru K Osaka A Large-scale preparation of ordered titania nanorods with enhanced photocatalytic activity Langmuir 2005 9 6995 7002 10.1021/la0500272 16008414

[B9] Wu YH Long MC Cai WM Dai SD Chen C Wu DY Bai J Preparation of photocatalytic anatase nanowire films by in situ oxidation of titanium plate Nanotechnology 2009 9 185703 10.1088/0957-4484/20/18/185703 19420626

[B10] de Tacconi NR Chenthamarakshan CR Yogeeswaran G Watcharenwong A de Zoysa RS Basit NA Rajeshwar K Nanoporous TiO_2_ and WO_3_ films by anodization of titanium and tungsten substrates: influence of process variables on morphology and photoelectrochemical response† J Phys Chem B 2006 9 25347 25355 10.1021/jp064527v 17165981

[B11] Quan X Yang SG Ruan XL Zhao HM Preparation of titania nanotubes and their environmental applications as electrode Environ Sci Technol 2005 9 3770 3775 10.1021/es048684o 15952384

[B12] Liu YB Zhou BX Bai J Li JH Zhang JL Zheng Q Zhu X Cai WM Efficient photochemical water splitting and organic pollutant degradation by highly ordered TiO_2_ nanopore arrays Appl Catal B Environ 2009 9 142 148 10.1016/j.apcatb.2008.11.034

[B13] Xu C Song Y Lu LF Cheng CW Liu DF Fang XH Chen XY Zhu XF Li DD Electrochemically hydrogenated TiO_2_ nanotubes with improved photoelectrochemical water splitting performance Nanoscale Res Lett 2013 9 7 10.1186/1556-276X-8-7 24047205 PMC3973816

[B14] Wu JM Huang B Zeng YH Low-temperature deposition of anatase thin films on titanium substrates and their abilities to photodegrade rhodamine B in water Thin Solid Films 2006 9 292 298 10.1016/j.tsf.2005.10.066

[B15] Wu YH Long MC Cai WM Novel synthesis and property of TiO_2_ nano film photocatalyst with mixed phases J Chem Eng Chin Univ 2010 9 1005 1010

[B16] Hu A Zhang X Oakes KD Peng P Zhou YN Servos MR Hydrothermal growth of free standing TiO_2_ nanowire membranes for photocatalytic degradation of pharmaceuticals J Hazard Mater 2011 9 278 285 10.1016/j.jhazmat.2011.02.033 21377796

[B17] Kavan L O’Regan B Kay A Grätzel M Preparation of TiO_2_ (anatase) films on electrodes by anodic oxidative hydrolysis of TiCl_3_ J Electroanal Chem 1993 9 291 307 10.1016/0022-0728(93)85020-H

[B18] Lei Y Zhang LD Fan JC Fabrication, characterization and Raman study of TiO_2_ nanowire arrays prepared by anodic oxidative hydrolysis of TiCl_3_ Chem Phys Lett 2001 9 231 236 10.1016/S0009-2614(01)00263-9

[B19] Hosono E Fujihara S Kakiuchi K Imai H Growth of submicrometer-scale rectangular parallelepiped rutile TiO_2_ films in aqueous TiCl_3_ solutions under hydrothermal conditions J Am Chem Soc 2004 9 7790 7791 10.1021/ja048820p 15212522

[B20] Feng XJ Zhai J Jiang L The fabrication and switchable superhydrophobicity of TiO_2_ nanorod films Angew Chem Int Ed 2005 9 5115 5118 10.1002/anie.200501337 16021643

[B21] Cho IS Chen Z Forman AJ Kim DR Rao PM Jaramillo TF Zheng X Branched TiO_2_ nanorods for photoelectrochemical hydrogen production Nano Lett 2011 9 4978 4984 10.1021/nl2029392 21999403

[B22] Lin J Liu K Chen X Synthesis of periodically structured titania nanotube films and their potential for photonic applications Small 2011 9 1784 1789 10.1002/smll.201002098 21591252

[B23] Lu Y Yu H Chen S Quan X Zhao H Integrating plasmonic nanoparticles with TiO photonic crystal for enhancement of visible-light-driven photocatalysis Environ Sci Technol 2012 9 1724 1730 10.1021/es202669y 22224958

[B24] Peter LM Dynamic Aspects of Semiconductor Photoelectrochemistry Chem Rev 1990 9 753 769 10.1021/cr00103a005

[B25] Long MC Beranek R Cai WM Kisch H Hybrid semiconductor electrodes for light-driven photoelectrochemical switches Electrochim Acta 2008 9 4621 4626 10.1016/j.electacta.2008.01.077

[B26] Abrantes LM Peter LM Transient photocurrents at passive iron electrodes J Electroanal Chem Interfacial Electrochem 1983 9 593 601 10.1016/S0022-0728(83)80238-1

[B27] Brusa MA Grela MA Experimental upper bound on phosphate radical production in TiO_2_ photocatalytic transformations in the presence of phosphate ions Phys Chem Chem Phys 2003 9 3294 10.1039/b302296j

[B28] Jiang DL Zhang SQ Zhao HJ Photocatalytic degradation characteristics of different organic compounds at TiO_2_ Nanoporous film electrodes with mixed anatase/rutile phases Environ Sci Technol 2007 9 303 308 10.1021/es061509i 17265963

